# Ucuùba (*Virola surinamensis*) Fat-Based Nanostructured Lipid Carriers for Nail Drug Delivery of Ketoconazole: Development and Optimization Using Box-Behnken Design

**DOI:** 10.3390/pharmaceutics11060284

**Published:** 2019-06-17

**Authors:** Rayanne R. Pereira, Matteo Testi, Francesca Rossi, Jose O. C. Silva Junior, Roseane M. Ribeiro-Costa, Ruggero Bettini, Patrizia Santi, Cristina Padula, Fabio Sonvico

**Affiliations:** 1 Pharmaceutical Sciences Faculty, Federal University of Para, 66075-110 Belem, Brazil; rayannerocha@yahoo.com (R.R.P.); carrera@ufpa.br (J.O.C.S.J.); roseribeiro01@yahoo.com.br (R.M.R.-C.); 2 Food and Drug Department, University of Parma, 43124 Parma, Italy; matteo.testi1@studenti.unipr.it (M.T.); ruggero.bettini@unipr.it (R.B.); patrizia.santi@unipr.it (P.S.); cristina.padula@unipr.it (C.P.); 3 Institute of Materials for Electronics and Magnetism (IMEM), CNR—Italian National Research Council, Parco Area delle Scienze 37/A, 43124 Parma, Italy; francesca.rossi@imem.cnr.it; 4 Biopharmanet-TEC, University of Parma, 43124 Parma, Italy

**Keywords:** Amazonian fat, Ucuùba fat, Box Behnken Design, solid lipid nanoparticles, antifungal therapy, onychomycosis

## Abstract

Ucuùba fat is fat obtained from a plant found in South America, mainly in Amazonian Brazil. Due to its biocompatibility and bioactivity, Ucuùba fat was used for the production of ketoconazole-loaded nanostructured lipid carriers (NLC) in view of an application for the treatment of onychomycosis and other persistent fungal infections. The development and optimization of Ucuùba fat-based NLC were performed using a Box-Behnken design of experiments. The independent variables were surfactant concentration (% *w*/*v*), liquid lipids concentration (% *w*/*v*), solid lipids concentration (% *w*/*v*), while the outputs of interest were particle size, polydispersity index (PDI) and drug encapsulation efficiency (EE). Ucuùba fat-based NLC were produced and the process was optimized by the development of a predictive mathematical model. Applying the model, two formulations with pre-determined particle size, i.e., 30 and 85 nm, were produced for further evaluation. The optimized formulations were characterized and showed particle size in agreement to the predicted value, i.e., 33.6 nm and 74.6 nm, respectively. The optimized formulations were also characterized using multiple techniques in order to investigate the solid state of drug and excipients (DSC and XRD), particle morphology (TEM), drug release and interactions between the formulation components (FTIR). Furthermore, particle size, surface charge and drug loading efficiency of the formulations were studied during a one-month stability study and did not show evidence of significant modification.

## 1. Introduction

Onychomycosis is the most common disease of the nail plate and nail bed [1,2,3]. Several antifungal agents are used in the treatment of onychomycosis, among them ketoconazole, an imidazole derivative that acts by blocking the synthesis of ergosterol. Oral treatment of onychomycosis with ketoconazole provides a cure rate of 50% to 70% in fingernail and 15% to 30% in toenail infections [4,5]. However, oral treatment with ketoconazole presents several disadvantages, such as long duration, drug interactions, systemic side effects, and high rates of recurrence. As a consequence, the marketed oral formulation (Nizoral, Janssen, Raritan, NJ, USA) was discontinued in 2013 for the treatment of fungal infections. Topical treatment appears safer and multiple topical preparations containing 2% ketoconazole in the form of a gel, cream or lotions are currently on the market. However, the topical treatments available have the disadvantage of low drug permeation through the nail [4,6,7].

Nanostructured lipid carriers (NLC) are colloidal dispersions in which the dispersed phase is composed of both solid and liquid lipids stabilized by an emulsifier [8,9]. Lipid nanoparticles have been extensively used for topical application, since their lipid matrix can interact with the *stratum corneum*, promoting drug delivery to the skin. Moreover, lipid nanoparticles have been shown to form an occlusive layer on the skin surface significantly increasing tissue hydration and drug skin permeation [10,11,12]. Similarly, it has been shown that lipid nanocarriers are increasing nail plate hydration enhancing drug penetration [13].

The type of solid lipid selected for the production of NLC has a pivotal role, because it affects a number of physicochemical and biopharmaceutical properties of the particles, such as surface charge, melting point, drug loading and release properties among others [14,15]. Plant derived fats are a viable option, since they are made of fatty acids important for maintaining the homeostatic balance of the skin and its annexes [16,17]. Ucuùba butter is extracted from the seeds of *Virola surinamensis*, a tree found mainly in the Amazon river basin, but present in an area that encompasses all of Central America and part of South America. Traditional uses of this fat span from the production of candles to medical soaps, but Ucuùba butter can be found also as a component of moisturizing creams and shampoos in the Brazilian cosmetic market [16,18]. The main fatty acids present in Ucuùba fat are myristic and lauric acid, while oleic, palmitoleic, linoleic acids are present at lower concentrations [19]. In addition, the presence in the leaves, root, bark and seeds of *Virola surinamensis* of phenylpropanoids, propiophenones, lignans, neolignans, polyketides and flavonoids with anti-oxidant, anti-inflammatory and anti-mycotic action has been reported in other studies and has elicited interest in the pharmaceutical properties of this plant [20,21,22].

In several studies related to the optimization of NLC production it was observed that the relative concentration of surfactant and lipids strongly affects the stability of the formulation [23]. The physical-chemical and biopharmaceutical characteristics of NLC, such as size, entrapment efficiency (EE), physical stability, drug release and morphology, are also strongly influenced by the choice and amount of lipids and surfactant [23,24,25]. Thus, an optimization of NLC production with the aid of design of experiment could be carried out with the objective of modulating the amount of liquid and solid lipids and surfactant to obtain the most desirable properties in terms particle size, entrapment efficiency and stability. Response surface method (RSM) is one of the prevalent approaches in the design of the experiments, which involves the use of different experimental designs to generate polynomial mathematical relationships mapping of the response over the experimental domain with the aim to predict the outcome of a process or select the optimal process parameters [26]. Box-Behnken design (BBD) is a type of RSM and is an independent, rotatable or nearly rotatable, quadratic design with the process variable combinations at the midpoints of the edges of the process space and its center. A significant advantage of BBD is that it is more cost effective compared to other techniques, such as Central Composite Design, 3-levels factorial design and D-optimal design, as it requires fewer experimental runs and less time for process optimization [26,27].

We hypothesized that Ucuùba fat, on account of its physicochemical and biological properties, could be a suitable lipid excipient for the production of NLC loaded with ketoconazole. Thus, the objective of this work was to obtain and optimize ketoconazole-loaded NLC production by high pressure homogenization using Ucuùba fat in the lipid phase. The design of experiment was then applied to select the optimal conditions for the production of Ucuùba fat NLC in terms of encapsulation efficiency (EE), particle size and polydispersity index (PDI) for use as a nail drug delivery system.

## 2. Materials and Methods

### 2.1. Materials

Virgin Ucuùba fat (*V. surinamensis)* was provided by Amazon Oil Industry (Ananindeua, Brazil). D-α-Tocopheryl polyethylene glycol 1000 succinate (TPGS 1000) was supplied by ISOCHEM (Gennevilliers, France). Propylene glycol monocaprylate (type II) (Capryol™ 90) was acquired from Gattefossé (Saint-Priest, France). Ketoconazole was purchased from Galena (São Paulo, Brazil). All other reagents were of analytical purity grade. Ultrapure (0.055 µS/ cm, TOC 1 ppb) was obtained with a Purelab Pulse + Flex Ultrapure water system (Elga Veolia, Milan, Italy).

### 2.2. Ucuùba Fat NLC Preparation

Ucuùba fat NLC were prepared using an emulsification, homogenization and solidification technique. The lipid phase was prepared by melting Ucuùba fat (T_m_ ~42 °C) at 10 °C above the lipid melting point and by mixing the liquid lipid with TPGS 1000 and Capryol™ 90. Ketoconazole was added to the lipid phase in the amount necessary to attain 0.5% *w*/*v* concentration in the final preparation. Then, the aqueous phase, preheated at 70 °C, was added gradually into the lipid phase under continuous magnetic stirring. Subsequently, a pre-emulsion was prepared using (Ultra-Turrax^®^ TP18/10, IKA Werke GmbH&Co., Staufen, Germany) operated at 10,000 rpm for 1 min. Finally, the pre-emulsion was processed using laboratory High Pressure Homogenizer (HPH) (Panda Plus 2000, GEA Niro Soavi S.p.A., Parma, Italy) at 70 °C for 10 successive cycles at a pressure of 500 bar. Nanoparticles were then left to cool to ambient temperature.

### 2.3. Experimental Design

A three-level three-factor Box-Behnken design (BBD) was applied for the optimization of the NLC production process using the statistical package Design-Expert^®^ Software (version 11, Stat-Ease Inc., Minneapolis, MN, USA). To this purpose, we selected three input factors, or critical process parameters (CPPs); namely, the concentration of the surfactant (TPGS), the concentration of liquid lipid (Capryol™ 90) and the concentration of the solid lipid (Ucuùba fat). These input factors were set at three levels, i.e., low, middle and high values, indicated conventionally by −1, 0 and +1, respectively. The design, including five repetitions of the central point, consists of a total of 17 experiments. The measured responses, or critical quality attributes (CQAs), were particle size (Y_1_), polydispersity index (PDI) (Y_2_) and encapsulation efficiency (EE) (Y_3_) (see Table 1).

For each CQA, the influence of the input factors and their interactions on the responses can be described for the non-linear quadratic model generated by the design as:

(1) $$Y=A_{0}+A_{1}X_{1}+A_{2}X_{2}+A_{3}X_{3}+A_{4}X_{1}X_{2}+A_{5}X_{2}X_{3}+A_{6}X_{1}X_{3}+A_{7}X_{1}^{2}+A_{8}X_{2}^{2}+A_{9}X_{3}^{2},$$

in which *Y* is the predicted response, *A*_0_ is the intercept, *A*_1_–*A*_9_ are the regression coefficients values. *X*_1_, *X*_2_ and *X*_3_ are the independent variables, the concentration of the surfactant, the concentration of liquid lipid and the concentration of lipid solid, respectively. The terms (*X*_1_*X*_2_, *X*_2_*X*_3_ and *X*_1_*X*_3_) and (*X_i_*^2^$X_{i}^{2}$ where *i*= 1,2 and 3) represent the interactions of the factors and the quadratic terms respectively.

### 2.4. Ucuùba Fat NLC Characterization

#### 2.4.1. Particle Size Distribution

Ucuùba fat NLC were characterized by dynamic light scattering using a Zetasizer Nano ZS (Malvern Pananalytical, Malvern, UK) applying Non-Invasive Back-Scatter (scattering angle 173°) at the temperature of 25 °C. NLC were diluted (1:100) with ultrapure water prior to measurements. Optimized NLC were measured at different days (0, 1, 7, 15 and 30 days). Average particle size (Z-average) and polydispersity index (PDI) obtained through the cumulants analysis of scattering data by the instrument software were measured in triplicate and results were expressed as mean and standard deviation.

#### 2.4.2. Zeta Potential

The zeta potential, representative of particle surface electrostatic charge, was determined by electrophoretic mobility (Zetasizer Nano ZS, Malvern, Pananalytical, Malvern, UK). The samples were diluted in 10 mM NaCl (1:500). Helmoltz-Smoluchowski equation was employed for the calculation of zeta potential by the instrument software.

#### 2.4.3. Encapsulation Efficiency (EE)

The content of ketoconazole in the nanoparticles was determined by the high-performance liquid chromatography (HPLC). The analysis was carried out on a Shimadzu chromatography system consisting of an autosampler (Model 542, ESA Biosciences, Chelmsford, MA, USA), a pump (LC-10AS, Shimadzu, Kyoto, Japan) and a UV-VIS detector (SPD-10 A, Shimadzu, Kyoto, Japan). A reverse phase C18 column (YMC-Pack ODS-AQ, 250 x 4.6 mm, particle size 5 µm, pore size 120 Å, YMC Co. Ltd., Kyoto, Japan) was employed for chromatographic separation in combination with a mobile phase consisting of a 75:25 (*v*/*v*) mixture of 0.5% *w*/*v* triethanolamine in methanol and 0.2% w/v ammonium acetate (pH 5). HPLC analyses were carried out at a flow rate of 0.8 mL/min, detection wavelength of 240 nm at 25 °C. Sample injection volume was 20 µL and sample elution time was 10 min.

The linearity of response for ketoconazole was verified in the range of 1−3 μg/mL (*R*^2^ = 0.999). Limit of detection and limit of quantification were 0.18 and 0.56 μg/mL, respectively.

The encapsulation efficiency (EE) of nanoparticles was determined by an indirect method. The amount of ketoconazole precipitated or present in agglomerates was quantified and subtracted from the total amount of drug present in the whole NLC dispersion. The drug in the solution (aqueous phase) was not taken into account, considering the poor aqueous solubility of ketoconazole [28]. Firstly, a precise volume of Ucuùba fat NLC preparation was dispersed in the mobile phase in order to extract the drug and quantitate the total drug present in the formulation by HPLC analysis. The second step consisted of determining the amount of drug precipitated in the form of crystals or particle agglomerates during the nanoparticles production. In order to separate the drug precipitated, the formulation was centrifuged at 9,500× *g* at 25 °C for 15 min (Model D3024, Scilogex, Rocky Hill, CT, USA). The supernatant was discarded without importing the pellet that was then solubilized in the mobile phase, and analyzed by HPLC.

The EE was calculated using Equation (2) and drug loading efficiency (*DL* %) were calculated using the Equation (3) [29],

(2) $$EE\;\left(\%\right)=\frac{\left(Total\;drug-Precipitated\;drug\right)}{Total\;drug}\times 100,$$

(3) $$DL\;\left(\%\right)=\frac{Total\;drug}{Total\;drug\;in\;feed}.$$

#### 2.4.4. Nanoparticles Morphology

Transmission Electron Microscopy (TEM) analysis of the NLC produced was carried out using a JEM 2200-FS microscope (JEOL Ltd., Tokyo, Japan) operated at 80 kV. For sample preparation, a drop of the suspension was deposited on formvar/carbon coated copper grids (300 mesh, Electron Microscopy Sciences, Holfield, PA, USA). After 60 s the excess was gently dried with filter paper and the grid was stained using a drop of 2% *w*/*v* of uranyl acetate solution (Sigma Aldrich, St. Louis, MO, USA) for 120 s. The staining solution was gently eliminated with filter paper and the grid was rapidly dipped in particle-free ultrapure water to further eliminate loosely bound material and excess staining. The images were processed with Digital Micrograph software (Gatan Inc., Pleasanton, CA, USA).

#### 2.4.5. In vitro Drug Release

*In vitro* release of ketoconazole was performed using the dialysis bag method [30]. The experiment was performed at 37° C using water:ethanol (50:50, *v*/*v*) as the release medium. The dialysis bag (MWCO 12,000 to 14,000 Da, Sigma Aldrich St. Louis, MO, USA) containing 1 mL of sample was placed in contact with 100 mL of release medium, assuring sink conditions, under moderate magnetic stirring. At predetermined time points (0, 1, 2, 3, 4, 5, 6, 7, 8 and 24 h), 300 μL of the release medium was withdrawn and replaced with the same volume of fresh medium. The concentration of ketoconazole was evaluated by HPLC with the same method described in Section 2.4.3. For comparison purposes, diffusion of unencapsulated ketoconazole from a hydroalcoholic mixture (50:50, *v*/*v*) at the same concentration was also evaluated. Three independent experiments were performed for each formulation.

Data were analyzed using mathematical modeling (KinetDS software, version 3.0, Kraków, Poland) in order to obtain a better understanding of the behavior of ketoconazole release from the NLC. The selection of the model that best described the release profile was based on the correlation coefficient (*r*).

#### 2.4.6. Differential Scanning Calorimetry (DSC)

Differential scanning calorimetry was performed using DSC823e equipment (Mettler-Toledo Schwerzenbach, Switzerland). All the samples, i.e., NLC, ketoconazole and excipients (mixtures Capryol™ 90/Ucuùba fat 3:1, 1:1 and 1:2) were prepared by introducing 1–5 mg of the sample into aluminum pans, sealed and double pierced. Thermal scans were recorded from 15 to 100 °C for the lipid mixtures and from 15 to 200 °C for the NLC samples at a heating rate of 10 °C/ min under dry nitrogen purge (80 mL/ min).

The samples of the NLC were air dried for 4h at room temperature for DSC analysis. Lipid mixtures were magnetically stirred at 200 rpm for 1 h at 70 °C for complete homogenization between lipids. The degree of crystallinity of mixtures of virgin Ucuùba fat and Capryol™ 90 was determined by calculating the crystallinity index (*CI*) from the heat of fusion using Equation (4) [31]:

(4) $$CI\left(\%\right)=\left(\frac{\Delta H_{\mathrm{mix}}}{\Delta H_{\mathrm{PL}}}\right)\cdot f_{\mathrm{mix}}\cdot 100$$

where *H*_mix_ is the enthalpy of fusion of the lipid mixture, *H*_PL_ is the fusion enthalpy of the pure lipid, and *f*_mix_ is the weight fraction of the solid lipid in the mixture.

Similarly, the *CI* (%) of the formulations was calculated using the Equation (5) [32]:

(5) $$CI\;\left(\%\right)=\left(\frac{\Delta H_{\mathrm{NLC}}}{\Delta H_{\mathrm{mix}\cdot \mathrm{concentration}\;\mathrm{lipids}}}\right)\cdot 100.$$

#### 2.4.7. X-ray Diffraction (XRD)

X-ray diffraction (XRD) of the NLC formulation and the Ucuùba fat was performed using a MiniFlex X-ray Diffractometer (Rigaku, Tokyo, Japan) using Cu K_α_ radiation (λ = 1.5418 Å) generated with 30 kV. About 200 mg of air dried NLC formulation and Ucuùba fat were loaded on the sample holder until it was completely full and then pressed with a glass slide in order to obtain a flat and homogeneous surface. The samples were analyzed from a starting angle θ of 2° to an end angle of 35° with a scanning rate of 1.5 °·min^−1^ (step size 0.5°).

#### 2.4.8. Infrared Spectroscopy

The absorption spectra of the excipients, i.e., Capryol™ 90, Ucuùba fat and ketoconazole, and NLC formulations, were analyzed using a Shimadzu spectrophotometer (IR Prestige 21, Kyoto, Japan) in the range 4000–1000 cm^−1^. The samples were dripped in tablets of potassium bromide and spectra recorded using 32 scans and 2 cm^−1^ resolution at room temperature [33].

## 3. Results

### 3.1. Design of Experiment

In the present study, a Box-Behnken design was applied to evaluate the interactions and the main quadratic effects of selected formulation parameters at different levels on three critical quality attributes, i.e., NLC particle size, polydispersity index and encapsulation efficiency. Table 2 summarizes the experimental results of the Box-Behnken design.

The mean particle size (response *Y*_1_) ranged from 23.9 nm (Experiment 16) to 90.1 nm (Experiment 8) depending on the variables level selected during production as indicated in Table 2. PDI values (response *Y*_2_) ranged from 0.248 (Experiment 2) to 0.558 (Experiment 11) indication that there were formulations with distributions varying from mostly unimodal to multimodal size distributions. The encapsulation efficiency (EE, response *Y*_3_) varied from 93.91% (Experiment 1) to 99.66% (Experiment 5) (see Table 2). The ratios between maximum and minimum values for responses *Y*_1_, *Y*_2_ and *Y*_3_ were found to be 3.76, 2.25 and 1.06, indicating no requirement of power transformation of values for the calculation of the model.

It was observed that the model of best fit was quadratic for all considered responses. The results of the analysis of variance (ANOVA) performed to investigate the models for the three responses (particle size, *Y*_1_; *PDI*, *Y*_2_; encapsulation efficiency, *Y*_3_) are shown in Table 3. The significance of the regression coefficients for the input factors was evaluated by the correspondent *p*-value (<0.05) calculated by ANOVA (see Table 3). The *p*-values were used as a tool to check the significance of each coefficient to understand the pattern of the mutual interactions between the selected process parameters. A *p*-value below 0.05 was considered an indication of a significant contribution of the factor (see Table 3). The fit of the model was evaluated by the coefficient of determination (*R*^2^).

#### 3.1.1. Particle Size

In the case of particle size, the value of the coefficient of determination (*R*^2^ = 0.9369) of the quadratic model indicated that only 6.31% of the total variations was not explained by the model (see Table 3).

**Figure 1:**
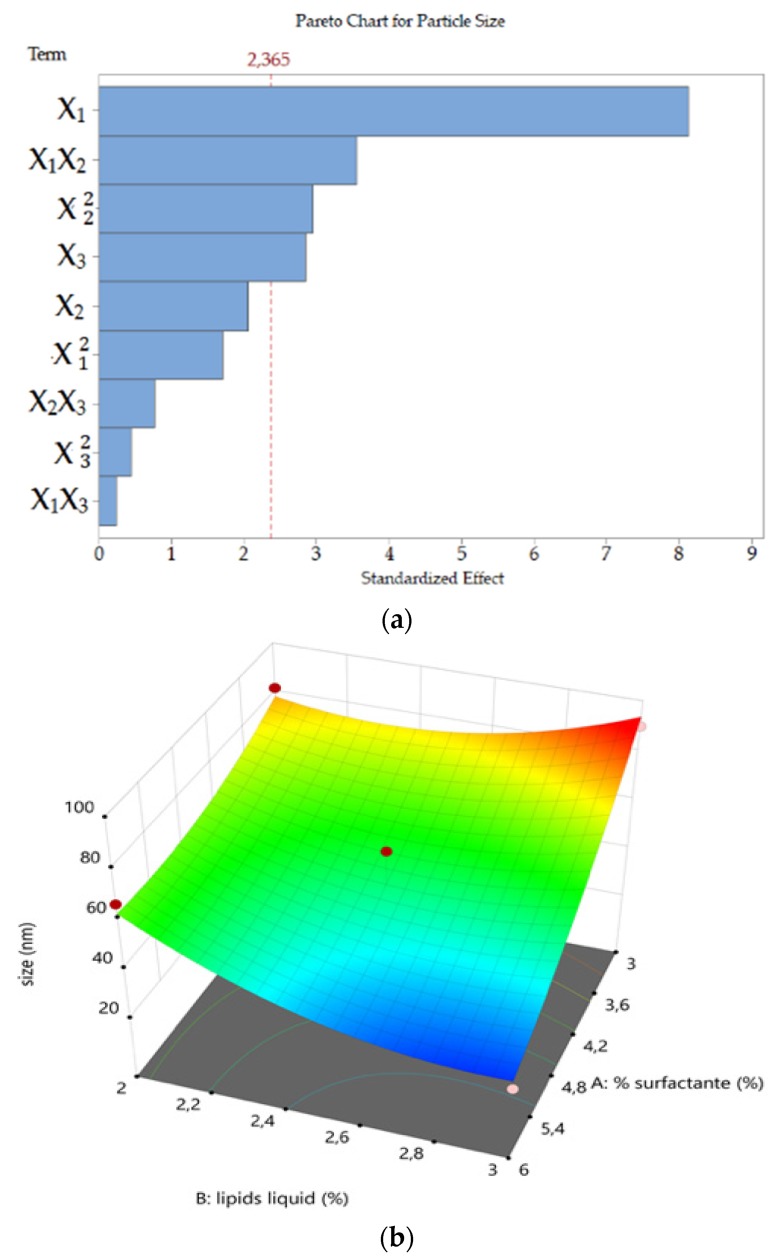

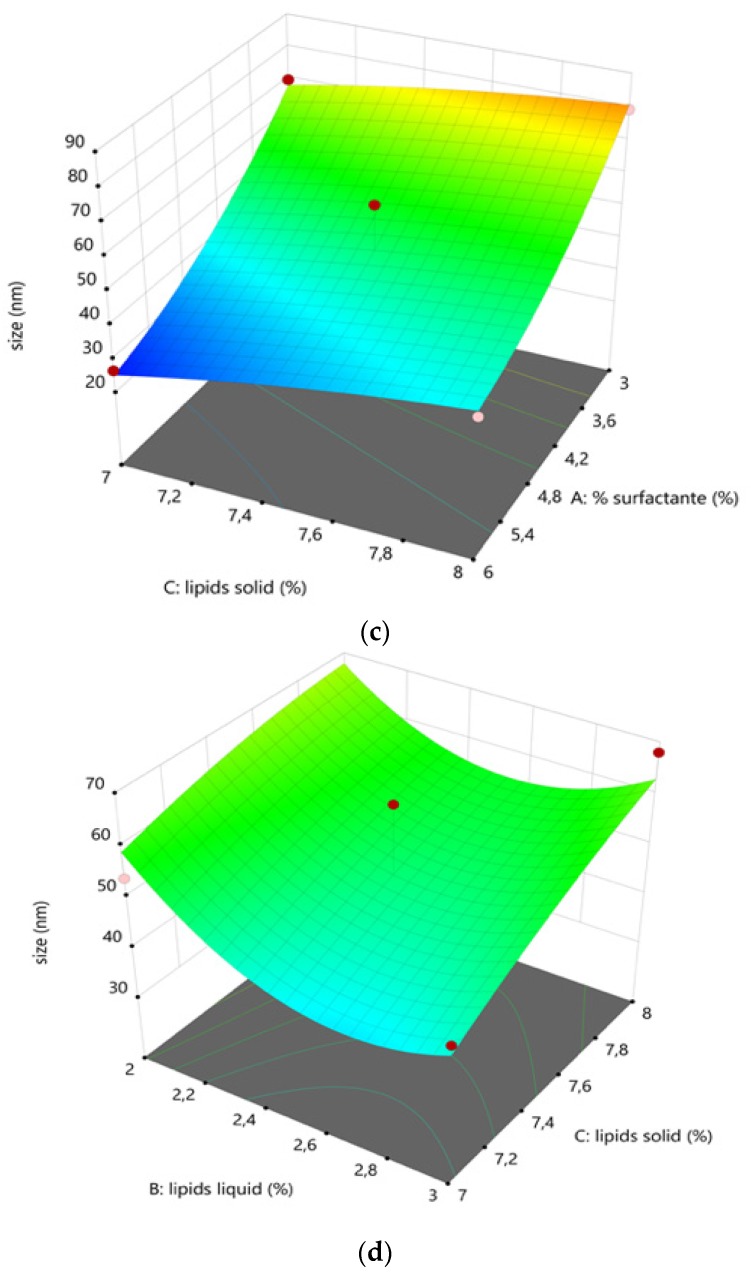
Pareto chart (**a**) and three-dimensional response surface plots showing the effect of (**b**) the concentration of liquid lipid and surfactant, (**c**) the concentration of surfactant and solid lipid and (**d**) the concentration of liquid lipid and solid lipid on nanostructured lipid carriers (NLC) particle size, respectively.

The quadratic model correlating the input factors to particle size resulted in the polynomial Equation (6) and allowed to design three response surface graphs (Figure 1) for the optimization lipid nanoparticle size,

(6) $$Y_{1}=49.49-20.54X_{1}-5.21X_{2}+7.19X_{3}-12.73X_{1}X_{2}+0.88X_{1}X_{3}+2.76X_{2}X_{3}+5.96X_{1}^{2}+10.29X_{2}^{2}-1.56X_{3}^{2}.$$

The concentration of surfactant (*X*_1_), liquid lipid (*X*${\;}_{2}^{2}$), solid lipid (*X*_3_) and interaction between surfactant and liquid lipid (*X*_1_*X*_2_) and were the factors significantly affecting (*p* < 0.05) the particle size (*Y*_1_) according to ANOVA (see Table 3 and Figure 1a). A positive coefficient value before a factor in the regression equation indicates that the response increases along with the factor and *vice versa*. The coefficient values show that particle size is strongly dependent on the concentrations of TPGS. In fact, as shown in Figure 1b, when maintaining the Ucuùba fat (solid lipid) concentration constant, lipid nanoparticles particle size decreased when the concentrations of Capryol™ 90 (liquid lipid) and TPGS (surfactant) increased (Figure 1b).

Similarly, by locking the concentration of Capryol™ 90 and varying the concentrations of TPGS and Ucuùba fat, it was observed that the lipid nanoparticles particle size decreases with the increase of the surfactant concentration and the decrease of the Amazonian fat concentration (Figure 1c). On the other hand, when the concentration of the surfactant was maintained constant and the concentration of lipids was varied (Figure 1d), fewer variations in terms of particle size were observed.

#### 3.1.2. Polydispersity Index (PDI)

For PDI, the value of the coefficient of determination of the quadratic model was *R*^2^ = 0.8477 indicating that 15.23% of the total variations was not explained by the model. This was higher when compared to the 6.31% the particle size related model (see all the values in Table 3).

The effect of the selected factors on PDI was described by the quadratic Equation (7),

(7) $$Y_{2}=0.400+0.087X_{1}-0.041X_{2}+0.004X_{3}-0.073X_{1}X_{2}+0.029X_{1}X_{3}-0.026X_{2}X_{3}-0.071X_{1}^{2}+0.033X_{2}^{2}-0.040X_{3}^{2}.$$

**Figure 2:**
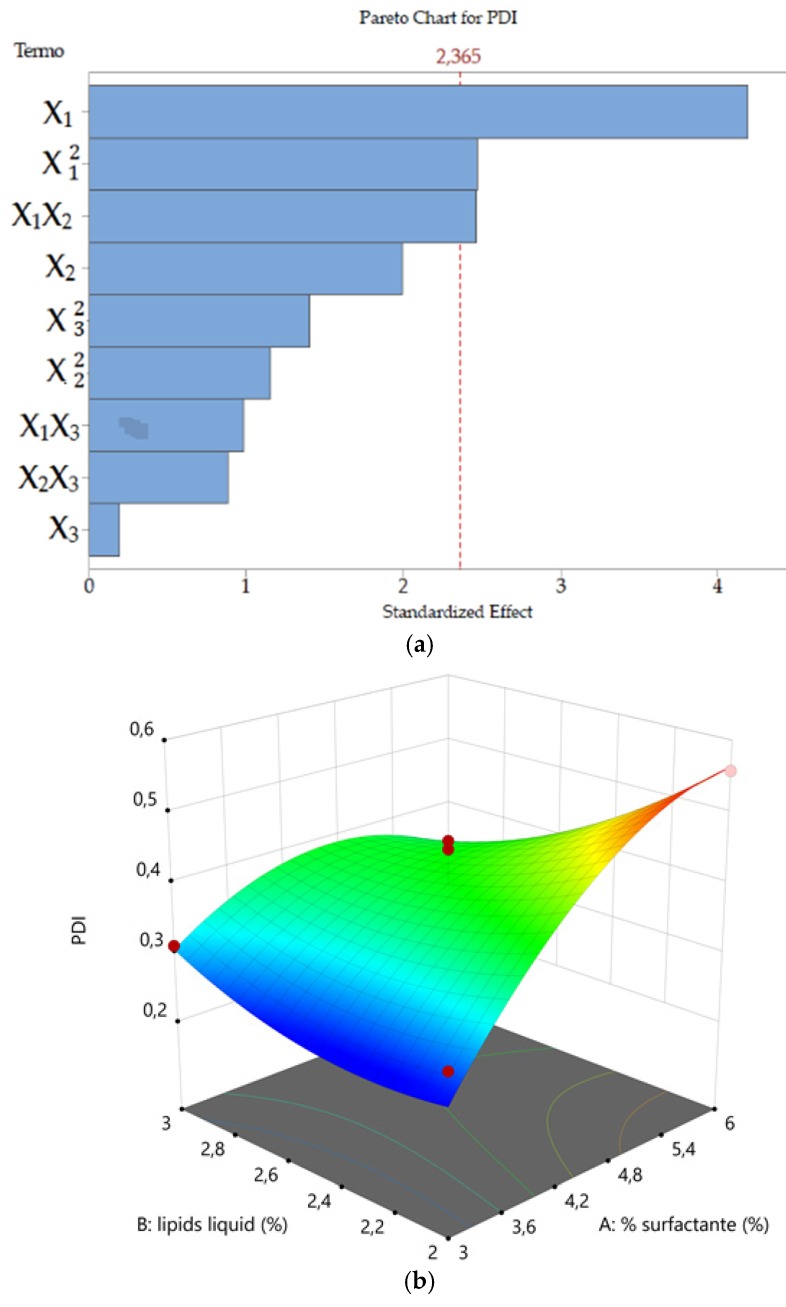

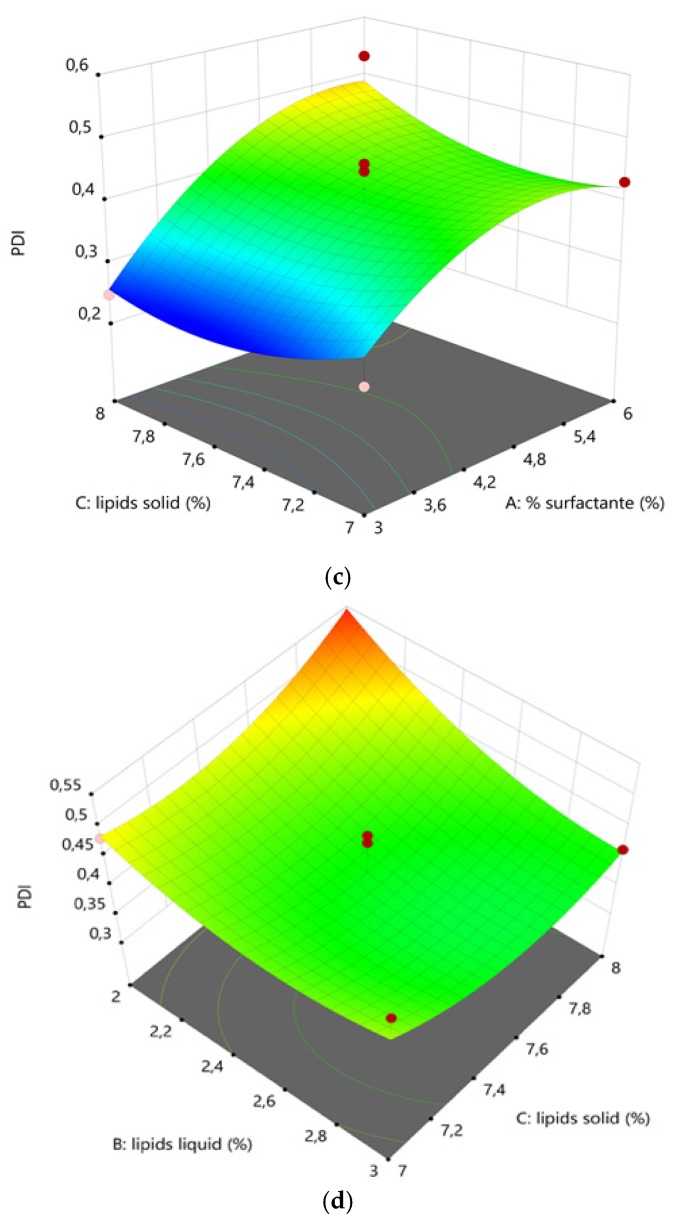
Pareto chart (**a**) and three-dimensional response surface plots showing the effect of (**b**) the concentration of liquid lipid and surfactant, (**c**) the concentration of surfactant and solid lipid and (**d**) the concentration of liquid lipid and solid lipid on NLC polydispersity index (PDI), respectively.

As shown in Figure 2a, the significant factors were the concentration of the surfactant (*X*_1_, *X*) and the combination of surfactant and liquid lipid concentration (*X*_1_*X*_2_). Figure 2 shows the response surface plots correlating the *PDI* to the factors investigated.

The concentration of surfactant (*X*_1_ and *X*${}_{1}^{2}$), and interaction between surfactant and liquid lipid (*X*_1_*X*_2_) and were the factors significantly affecting (*p* < 0.05) the PDI (*Y*_2_) according to ANOVA (see Table 3 and Figure 2a). Analyzing the response surfaces for PDI, it appeared that this nanoparticle attribute was again mainly influenced by the surfactant concentration (Figure 2b,c). However, differently from particle size, PDI steeply increased when the TPGS concentration was increased. The Ucuùba fat and Capryol™ 90 concentrations resulted less relevant for PDI values compared to their effect combined with surfactant variations, even if the PDI appeared to increase along with both the solid lipid and the liquid lipid concentrations. In any case, the response surface plot representing Ucuùba fat and Capryol™ 90 interaction, keeping constant TPGS concentration, resulted overall in a not significant PDI variation (Figure 2d).

#### 3.1.3. Ketoconazole Encapsulation Efficiency (EE)

**Figure 3:**
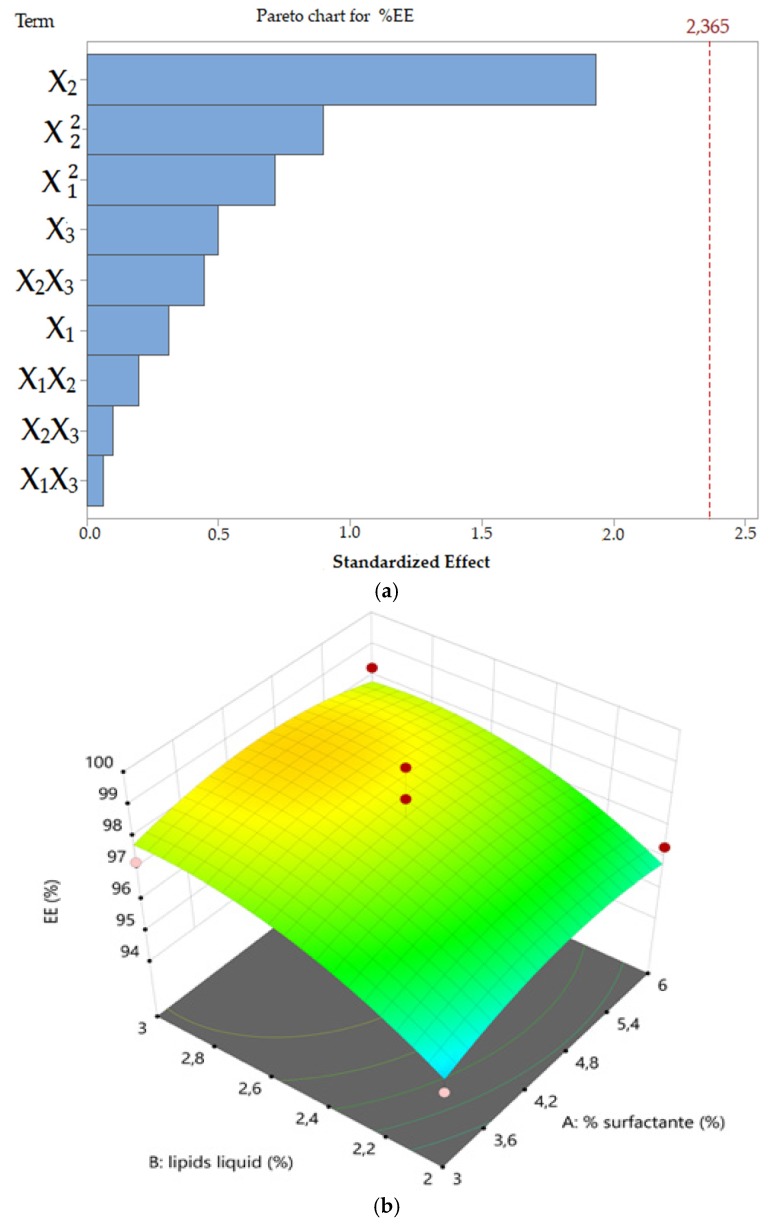

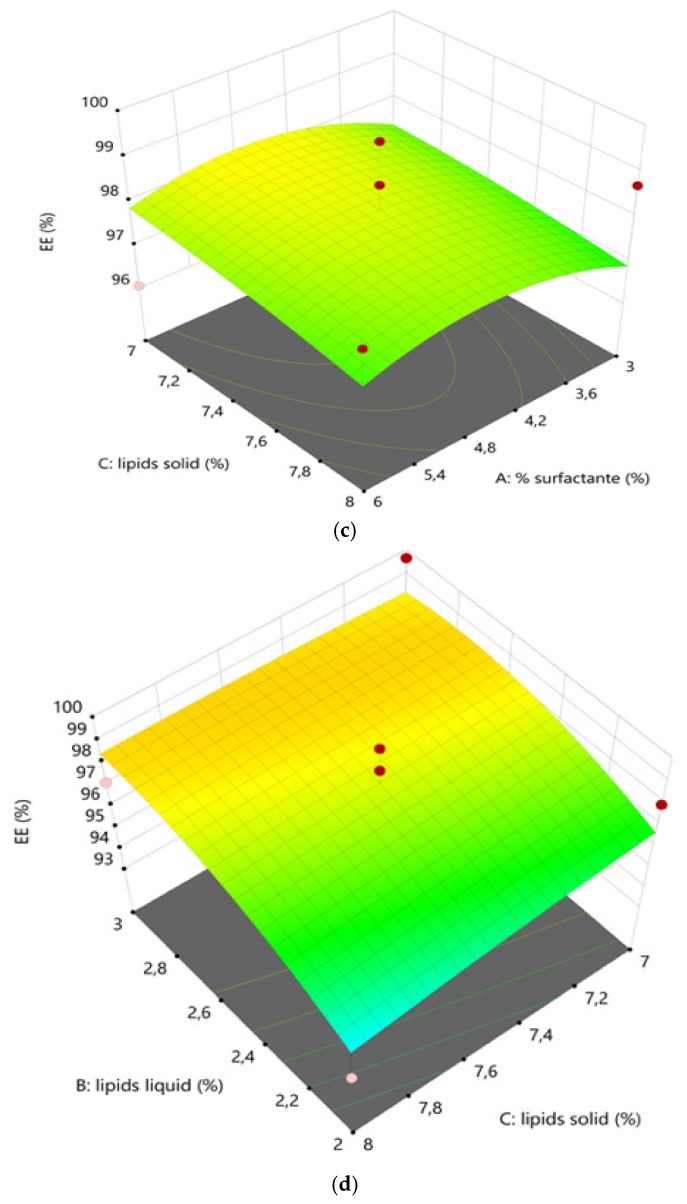
Pareto chart (**a**) and three-dimensional response surface plots showing the effect of the concentration of liquid lipid and surfactant (**b**), the concentration of surfactant and solid lipid (**c**) and the concentration of liquid lipid and solid lipid (**d**) on NLC ketoconazole encapsulation efficiency (EE), respectively.

The model correlating ketoconazole EE to the selected factors was not found to be significant (*R*^2^ = 0.4513, *p*-value 0.7392) (Figure 3). Within the present design space, it was not possible to achieve a significant difference in terms of encapsulation efficiency. However, it was evidenced that ketoconazole at the selected concentration of 0.5% w/v could be efficiently loaded into NLC obtained using TPGS surfactant, Capryol™ 90 as liquid lipid, and Ucuùba fat as solid lipid within the range of concentration tested (Figure 3).

#### 3.1.4. Formulation Optimization and Model Validation

A desirability function was applied to the models obtained to identify the process parameters required to prepare optimized formulations. The optimal formulation was based on the criteria of maximum EE percentage, minimum PDI and specific particle size. The actual values of the dependent variables at the optimal combination of factors suggested by the software were assessed to confirm the validity of the calculated optimal factors. In particular two optimized formulations were prepared using the model, one with smaller (30 nm, F 30) and a second with larger (85 nm, F 85) required particle size, respectively. Other requirements were minimum PDI and maximum EE. The composition of the optimized formulations is presented in Table 4. Formulation F 85 showed the highest residual value in terms of size (10.4 nm) and PDI (0.114), this value indicates how much the experimental value is far from the predicted value. The overall desirability value for these formulations (obtained using a desirability function that assigns numbers between 0 and 1 of the possible values each response with 0 representing a completely undesirable value and 1 representing the ideal response value) was 0.86 for F 85 and was 0.78 for F 30. The good correlation between the actual and predicted results indicates that BBD along with desirability function could be successfully used to optimize the nanoparticles manufacturing process.

**Figure 4:**
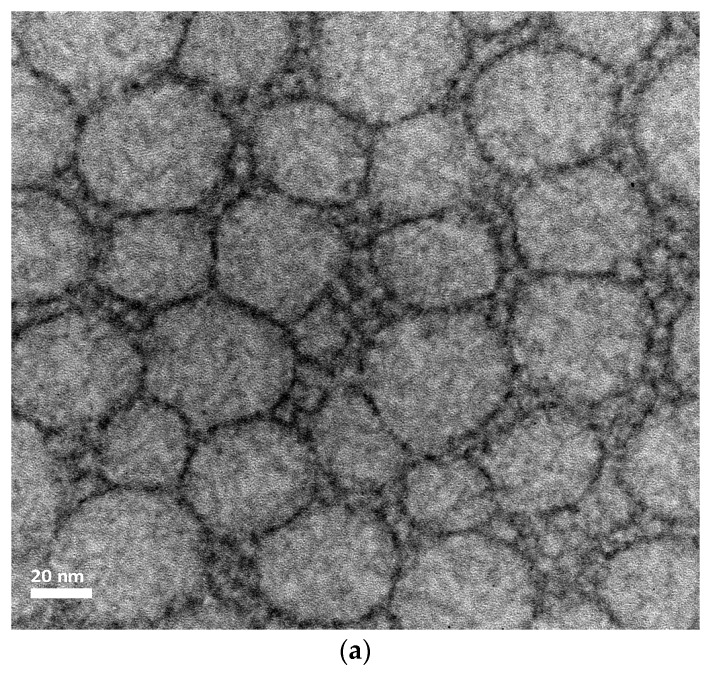

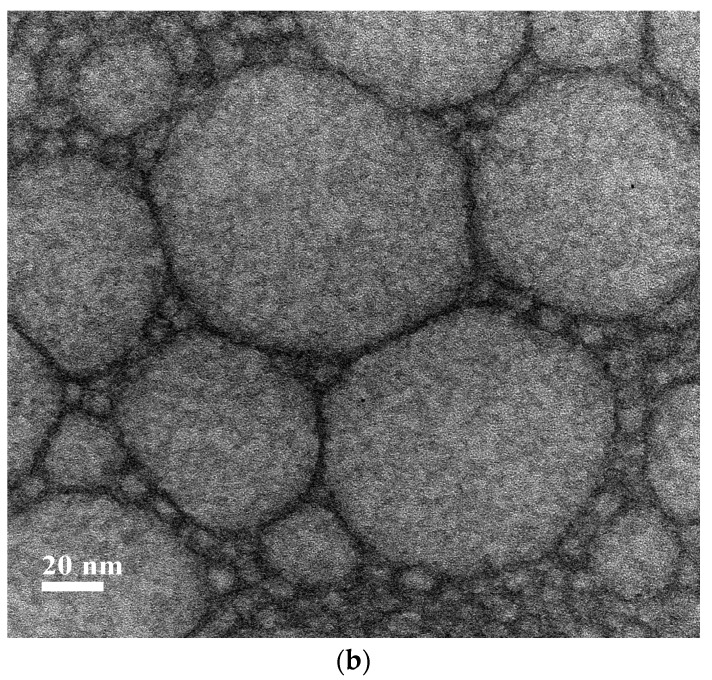
Representative TEM images of the optimized Ucuùba fat NLC formulations (**a**) F 30 and (**b**) F 85 (magnification 100,000×).

**Figure 5:**
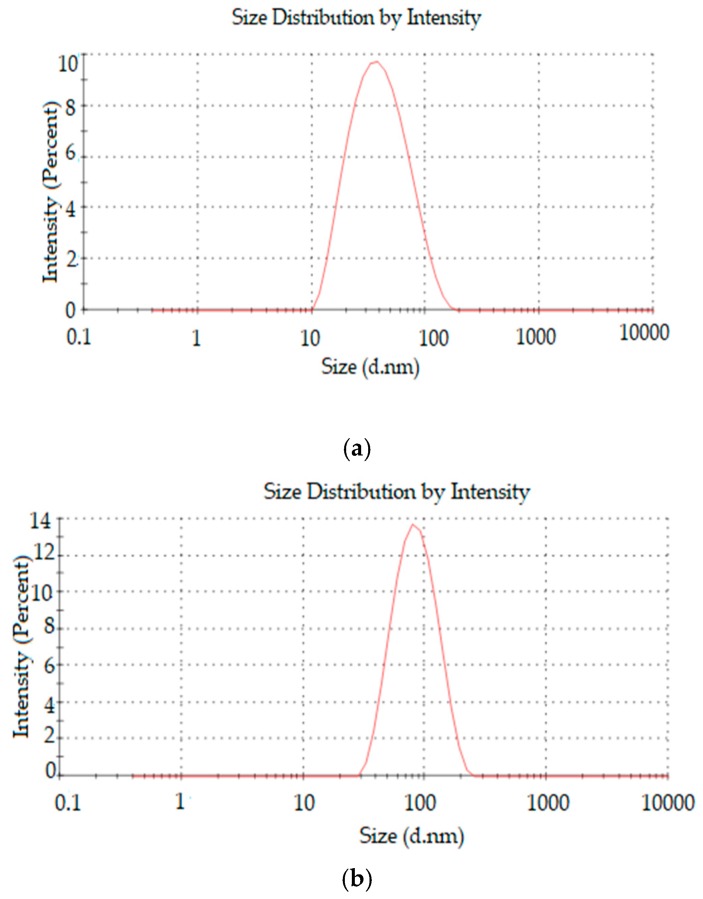
The particle size distribution of (**a**) F30 and (**b**) F85 by dynamic light scattering (DLS).

Figure 4 and Figure 5 present TEM images and dynamic light scattering (DLS) particle size distribution of the NLC optimized formulations F 30 and F 85, respectively.

The two NLC formulations displayed a globular shape, characterized by a spherical homogeneous lipid core surround by a surfactant corona. It can be observed from the images that the particle size confirmed the low polydispersity of NLC, as indicated by the relatively small PDI values obtained with the DLS measurements and by the relative particle size distribution graphs presented in Figure 5.

### 3.2. Stability Study of Ucuùba Fat NLC Prepared According to the Optimized Conditions

Ucuùba fat NLC prepared according to the optimized conditions were characterized immediately after preparation and up to 30 days of storage at room temperature. Results are shown in Table 5.

The average particle size did not change significantly during the 30 days of observation. Furthermore, the PDI of the optimized Ucuùba fat NLC were found to be consistently below the values 0.16 and 0.26 for F 85 and F 30 respectively (Table 5), indicating a relatively narrow particle size distribution and absence of agglomerates for up to 30 days storage. All formulations exhibited negative zeta potential, which was around −15 and −25 mV for F 30 and F 85, respectively and did not show significant changes during the 30 days storage (*p* > 0.05). Another parameter analyzed was the drug loading efficiency (DL%), calculated 24 h after NLC preparation, and again after 15 and 30 days. The drug loading efficiency value for F 30 was 85.6% on the first day after production, and 85.9% and 86% on days 15 and 30, respectively. For F 85, the DL% values on days 1, 15 and 30 were 86.7, 87.1 and 86.5%, respectively. Formulations were stable over time despite an initial drug loss probably occurring during the pre-emulsification and homogenization phase.

### 3.3. In Vitro Drug Release

**Figure 6:**
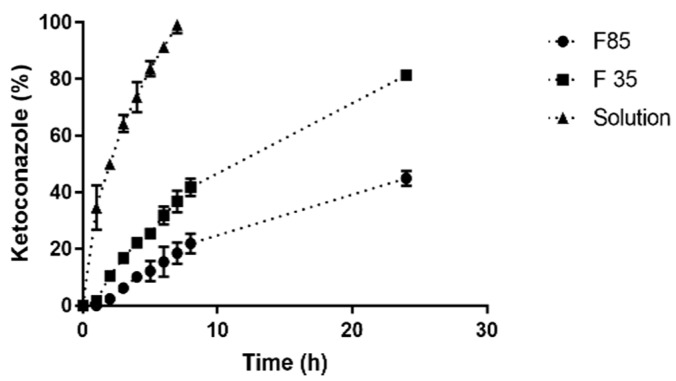
The release profile of ketoconazole from F 30 and F 85 using the dialysis bag method (*n* = 3, ±SD).

The in vitro release profiles of F 30 and F 85 were determined in a 24 h dissolution experiment (Figure 6). A solution of ketoconazole was used as a control in order to evaluate the barrier to drug diffusion determined by the presence of the dialysis membrane. The results showed that NLC provided a controlled release of ketoconazole. In fact, the percentage of drug released from F 30 and F80 after 24 h was 81.3 ± 0.64% and 45.0 ± 2.7%, respectively; while the solution diffusion out of the dialysis bag was already completed after 7 h. Differences in lipid phase composition (Capryol™ 90, Ucuùba fat and surfactant) of NLC not only provided differences in particle size, PDI and EE but also a modulation of drug release rate.

### 3.4. Solid State Analysis

#### 3.4.1. Differential Scanning Calorimetry (DSC)

**Figure 7:**
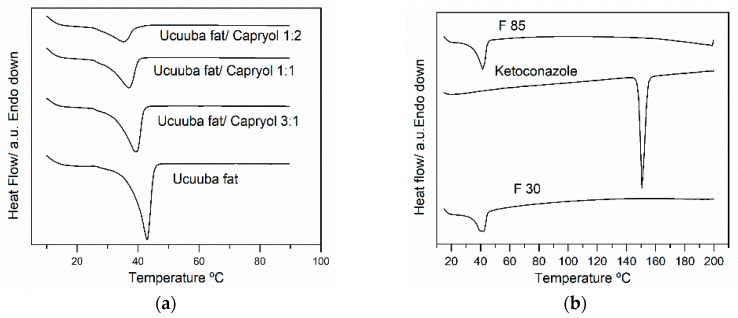
DSC traces of Ucuùba fat/Capryol™ 90 mixtures (**a**) and of the optimized Ucuùba fat NLC formulations F 30 and F 85 along with ketoconazole raw material (**b**).

DSC is widely used for investigation the solid state of drugs and excipients, such as lipids. In particular, due to the melting point identification and melting enthalpies determination, this technique highlights the modifications of the crystalline components of the formulations, complementing the XRD data (see Section 3.4.2.). The Ucuùba fat evidenced a melting point of 43 °C while the blends of Ucuùba fat with Capryol™ 90 showed lower melting points (see Figure 7a and Table 6). At the same time a reduction of the melting enthalpy was evident.

Table 6 shows the crystalline index value (*CI*) which decreases with the increase in the amount of Capryol™ 90. The data evidenced that the mixture with Capryol™ 90 affects Ucuùba fat crystalline lattice order contributing to increase disorder and causing the decrease *CI* and a shift in the melting point to a lower value. This last characteristic is desirable for the production of NLC, since a solid fat with less ordered lattice, reduces the probability of stability issues related to the expulsion of the entrapped active compound during storage as reported for some solid lipid nanoparticles (SLN) [28].

The DSC results presented in Figure 7b are related to ketoconazole and the two optimized formulations F 30 and F 85 after drying. It can be observed that only one endothermic peak appeared in the DSC traces obtained for both NLC formulations F 30 and F 85. For formulation F 30 a single melting peak at 41.86 °C and melting enthalpy of 49.13 J/g were observed during the heating process, attributed to lipid matrix solid-to-liquid transition. Similarly, for F 85 a single melting peak was evident at 41.59 °C, corresponding to enthalpy of −54.59 J/g, which was also related to the lipid matrix melting. Considering that ketoconazole showed an endothermic peak of fusion at 150.76 °C, it can be inferred that ketoconazole is molecularly dispersed in the lipid matrix and no detectable drug crystals are formed during the production of the NLC, even if the relatively low drug concentration makes the detection of crystalline ketoconazole difficult.

The CI values of the two optimized formulations were 68.64% and 75.65%, for F 30 and F 85, respectively (Table 6). The increased crystallinity of the F 85 formulation is probably due to the slight increase in the solid to liquid lipid ratio, but mainly to the reduction in surfactant concentration. It is also possible that the larger particle size favored the higher crystallinity of the lipid phase. The matrix solid state is probably another factor that led to different dissolution rates between F 30 and F 85 (see Figure 6).

#### 3.4.2. X-ray Diffraction (XRD)

**Figure 8:**
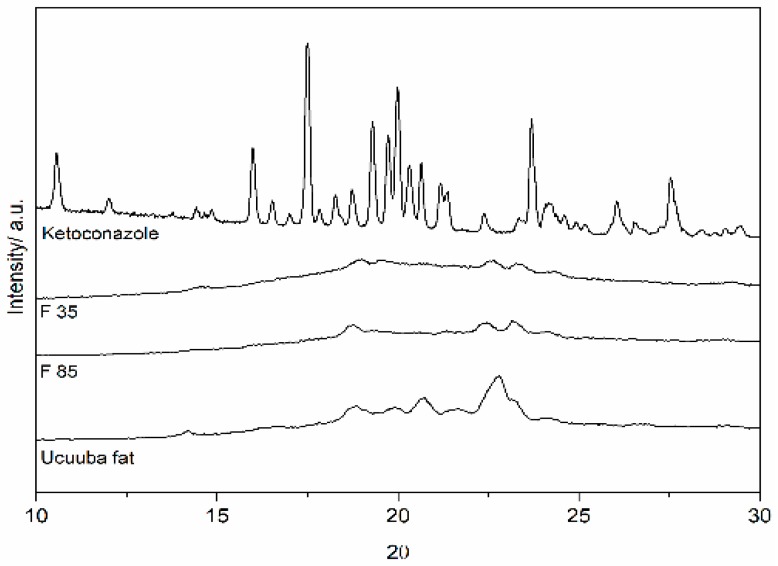
The X-ray diffraction spectra of Ucuùba fat and optimized Ucuùba fat NLC formulations F 30 and F 85.

Ucuùba fat is a mixture of mono-, di- and tri-triglycerides (trimyristin being the main component) characterized by a complex polymorphism that can be evidenced by X-ray diffraction. The diffractograms of the Ucuùba fat and the dried NLC formulations F 30 an F 85 are shown in Figure 8. The diffraction pattern of Ucuùba fat appears similar to the diffraction pattern of F 30 and F 85 (presence of peaks at 2 theta 18.8°, 22.6° and 23.5°), but the diffractograms of the NLC formulations show broader and less defined peaks indicating a solid state more amorphous than the virgin Ucuùba fat. This was expected considering the presence of the liquid lipid Capryol™ 90 in the NLC formulations which reduced the Amazonian fat organization as demonstrated by DSC data.

At the same time, it was observed that of the numerous peaks characterizing the diffraction pattern of ketoconazole crystalline powder (see Figure 8) none appeared in the diffractogram obtained for ketoconazole-loaded NLC formulations F 30 and F 85. This possibly indicates that ketoconazole is completely dispersed in the lipid matrix, even if due to the relatively low drug loading the presence of the crystalline drug would be difficult to detect.

#### 3.4.3. Furrier-Transform Infrared Spectroscopy (FTIR)

**Figure 9:**
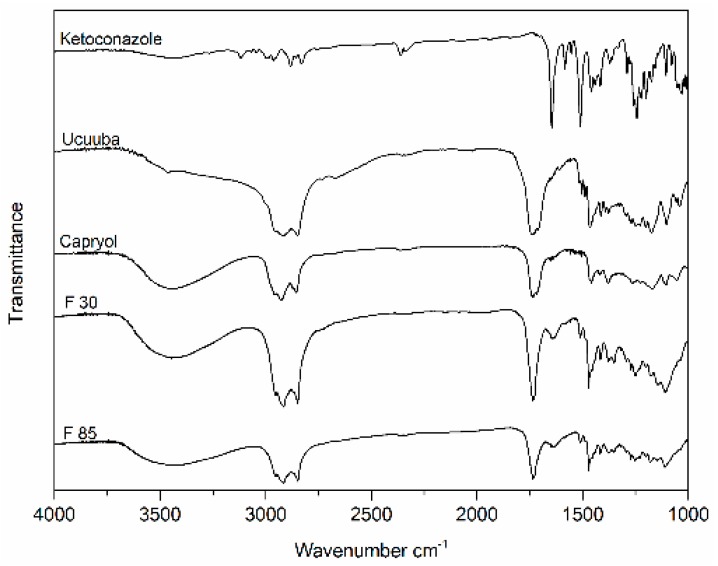
FT-IR spectra of ketoconazole, Ucuùba fat, Capryol™ 90 and of optimized Ucuùba fat NLC formulations F 30 and F 85.

FTIR is a valuable qualitative tool for characterization of the interaction of the components of pharmaceutical systems. FTIR analysis was performed for the main components of the NLC, i.e., Capryol™ 90, Ucuùba fat and ketoconazole, and the two Ucuùba fat NLC formulations (F 30 and F 85). The spectra are presented in Figure 9 and it appears clear that the FT-IR spectra of Ucuùba fat, Capryol™ 90 and NLC samples presented striking similarities and were almost superimposable. In these spectra, the high intensity peak at 1745 cm^−1^ corresponds to the vibration of carbonyl groups [33]. The vibration of the C–O bond of the ester bonds present in triacylglycerols is related to the sequence of peaks identified at 1232, 1165, 1118 and 1097 cm^−1^ [35]. The high intensity bands observed at 2922 and 2850 cm^−1^ are characteristic of the C–H stretching vibrations of lipids and correspond to olefinic (CH=CH) double bonds and methyl and methylene groups of fatty acid chains, respectively (Figure 9) [36]. Overall these bands, that are characteristic of the lipid nature of Ucuùba fat and Capryol™ 90, are also strongly represented in the FTIR spectra of the NLC formulations in which the lipid matrix is the defining component.

The FT-IR spectrum of pure ketoconazole shows at least three characteristic peaks at 1647, 1510 and 1242 cm^−1^, corresponding to the carbonyl (C=O), aromatic ring and C–N piperazine bonds stretching, respectively [37]. The spectrum of ketoconazole in the spectra of formulations has almost completely disappeared, suggesting an efficient drug entrapment in the solid lipid nanoparticle matrix. However, the drug loading is demonstrated by the presence of weak bands between 1600 and 1650 and around 1250 cm^−1^, which were not so evident in the FTIR spectra of the lipid excipients.

## 4. Discussion

The main purpose of this study was the development and optimization of Ucuùba fat NLC, using ketoconazole as a drug model for the treatment onychomycosis. Since the introduction at the beginning of the new millennium of the Quality-by-Design approach, the pharmaceutical sector has started using the design of the experiment as a statistical tool to maximize process knowledge and efficiently optimize product quality characteristics [38,39]. This approach has been applied to the manufacturing process of several dosage forms, including as tablets [40], pellets [41], semisolid formulations [42] and spray-dried powders to be used in dry powder inhalers [43].

Design of Experiment (DoE) was deemed suitable for the development and optimization of innovative and complex dosage forms, such as nanoparticles. In fact, scale-up manufacturing issues and poor process control have been indicated as some of the critical underlying problems that hampered the transfer of nanomedicines to the clinic [44]. At the same time, the potential of nanoformulations, such as lipid nanovectors, and their inclusion in oral [45,46] or transmucosal medicinal products have not yet been fully studied [47,48,49].

For these reasons, high-pressure homogenization, a robust and easy to scale up method, was selected for the manufacturing of NLC in this study [50] and a BBD was applied to understand and optimize of Ucuùba fat NLC manufacturing process. The BBD results in a mathematical model which describes as closely as possible effects of the factors and their levels on the critical quality attributes of the product over the whole design space, and also predicts the responses [51].

The high-pressure homogenization manufacturing process led to homogeneous, clear and transparent yellow-brownish dispersions. Particle size (*Y*_1_) analysis confirmed that it was possible to obtain dispersions below 100 nm over the experimental design space. Particle size was strongly influenced by TPGS and Ucuùba fat concentrations. The liquid lipid Capryol™ 90 influenced the particle size only in combination with TPGS (*X*_1_*X*_2_, *p* = 0.0092) probably because it acts as co-surfactant in this formulation.

In other studies, surfactant concentration was also a significant factor in decreasing particle size. Indeed, increasing the amount of surfactant in the system usually results in smaller particles until a saturation point is reached where the amount of surfactant does not affect the average particle size [25,52]. In particular, TPGS is a vitamin E derivative and a nonionic surfactant, it is able to stabilize nanoemulsions by lowering interfacial tension with steric repulsion between nanoparticles. As a consequence, bigger amounts of surfactant result in smaller particle size [53]. Interestingly, TPGS has recently been shown to release tocopherol and tocopherol succinate by enzymatic cleavage of ester bonds [54]. Alpha-tocopherol has been demonstrated to be able to increase amphotericin B activity against *Candida albicans* [55]. Therefore, the increase in surfactant concentration could not only contribute to particle size decrease, but high TPGS concentrations could result in enhanced antifungal activity of ketoconazole.

Although Capryol™ 90 and TPGS contributed to the decrease in particle size, Ucuùba fat acted in the opposite direction. It was observed, in this DoE, that Ucuùba fat content increase is always related to an increase in particle size (see Equation (6)). The physicochemical characteristics of Ucuùba fat can actually affect nanoparticles size. For example, the viscosity of the lipid phase influences the average particle size of the NLC during the pre-emulsion and homogenization stages at high pressure. The lower the viscosity of the two phases, the smaller the particles formed [56]. An increase in the amount of Ucuùba fat in the lipid phase increases the viscosity of the system and causes greater resistance to the shear forces applied during homogenization, resulting in larger particles.

PDI (*Y*_2_) values ranged from 0.248 to 0.558. This parameter reflects the homogeneity of the nanoparticle populations, values lower than 0.3 indicates relatively narrow particle size distribution and values even lower associated with monodisperse particles are generally desirable. On the contrary, higher values are associated with polydisperse particle size distribution [57]. In our DoE, formulations with high surfactant concentration showed PDI values above 0.4 and multimodal size distribution. The correlation among surfactant concentration and increasing PDI value could be explained when considering that an excess of TPGS tends to self-assemble into micelles. Furthermore, it is possible that during the Ucuùba fat NLC preparation with high pressure homogenizer the temperature increase could have decreased the TPGS critical micelle concentration (CMC) and favored the production of different micelle populations leading to PDI increase [29].

Concerning ketoconazole EE (*Y*_3_) was found to range from 93.91 to 99.66%. In this study, the *EE* was not found to be dependent on the process parameters explored. This result was probably obtained because the percentage of ketoconazole was maintained constant in all formulations (0.5% *w*/*v* ketoconazole) and Ucuùba fat NLC offered sufficient lipid matrix to fully solubilize and encapsulate the ketoconazole inside the nanoparticles. The lipid phase in NLC was a mixture of solid and liquid lipids and this peculiar feature is the reason for its higher encapsulation efficiency and better storage stability when compared to solid lipid nanoparticles (SLN) [58]. In particular, the DSC thermograms of Ucuùba fat was found to be affected by the mixing with Capryol™ 90 and it was observed that increasing the ratio of Capryol™ 90 a broadening of the melting peak of the solid fat was observed, possibly indicating the formation of several polymorphic forms. Similarly, the effect of the addition of α-tocopherol on the crystalline organization of Compritol^®^ ATO 888, for NLC production was studied and it was observed that the addition of 30% α-tocopherol decreases the melting point of Compritol from 71 to 50.3 °C. The authors attributed this melting point decrease to the appearance of less organized polymorphic arrangements in the solid lipid, due to the addition of α-tocopherol [59].

Drug release studies are routinely conducted to examine the ability of nanoparticles to modulate drug delivery. Ketoconazole release profile from F 30 and F 85, evidenced a faster release for the former formulation, suggesting that the size of the nanoparticle influences the rate of drug release by NLC. Small particles develop a larger surface area allowing for faster drug release [30,60]. Another factor at play probably was the lipid matrix crystallinity of the nanoparticles, since the higher fat content of Ucuùba fat in F 85 formulation together with low surfactant content determined a higher lipid matrix crystallinity index value for F 85 which may have contributed to the slower release of ketoconazole.

In general for NLC, the release of the drug is attributed to the components degradation, lipid matrix erosion or drug diffusion from nanoparticles [60]. Sometimes NLCs present a release characterized by two phases: An immediate release (or burst release) followed by a sustained release [60,61]. In this study, it was observed that the release profile of the formulation F 85 does not present an immediate release, while for F 30 the situation was different. Using KinetDS software the drug release data were fitted using several kinetic models in order to better understand the mechanism of ketoconazole release for both F 30 and F 85 formulations. The release profile obtained for F 85 NLC was best fitted by Higuchi equation (*R*^2^ = 0.9801), that usually describes the diffusion of the drug from homogenous and granular matrix systems [62]. This suggests that ketoconazole release is mainly due to drug diffusion from the lipid matrix and partitioning between the lipid and the aqueous phase [63]. Release data for F 30 NLC were fitted instead using the Weibull equation with lag time (*R*^2^ = 0.9959). The release mechanism for F 30 NLC is then described best by an initial burst release phase, probably due to the released drug being adsorbed on the surface of nanoparticles, followed by a typical sustained drug-release behavior.

The measurements of lipid crystallinity and its modification are strongly connected with drug release and drug incorporation in lipid-based drug delivery systems. In order to study the lipid matrix crystalline organization, XRD is a widely used technique also for the investigation of SLN and NLC [64]. The lipid polymorphism is a property that allows the triglycerides to exist in the form of several crystalline structures characterized by different molecular packing [65]. The three most common types of structure for triglycerides are hexagonal, orthorhombic and triclinic, which are usually referred to as polymorphic forms α, β’and β, respectively. The type of crystalline form depends on the composition of the lipid mixture and on the molecular structure of each lipid, as well as on the environmental conditions during processing and storage (cooling rate, for example). For the Ucuùba fat-based NLC, the sharp peaks identified at 18.9°, 22.6° and 23.2°, correspond to short molecular spacings at 0.46, 0.393 and 0.382 nm, indicative of β and β’ structures, respectively [65,66].

Zeta potential values are often used to predict the stability of nanoparticles. In fact, this parameter reflects the charge of the surface of nanoparticles in suspensions and high zeta potential values indicate high repulsive forces between particles, preventing them from forming large particle size agglomerates [10,57,67,68]. In general, nanomaterials with zeta potential above 30 mV are considered physically stable [34,69]. However, in our formulations the zeta potential absolute values were found to be smaller than 30 mV. However, these systems contain a steric stabilizer, i.e., the non-ionic and PEGylated surfactant TPGS, and as a consequence, the absorption of the surfactant on nanoparticle surface decreases the zeta potential, due to the shift of the shear plane of the electrical double layer existing around each particle [10]. However, the surfactant layer provides a steric contribution to particle stability, due to the presence of the hydrophilic PEG polymer chains [47].

## 5. Conclusions

Nanostructured lipid carriers containing an Amazonian fat were manufactured using a high-pressure homogenizer and the process was studied and optimized using a design of experiment approach. Results obtained allowed to build a predictive mathematical model showing that among the critical process parameters studied surfactant and Ucuùba fat concentrations were the most significant factors for the determination of the nanoparticles critical quality attributes, i.e., average particle size and particle size distribution in terms of PDI. The encapsulation efficiency was found to be above 90% for all the experimental design space and the factors investigated did not significantly affect its value in this model.

Using the model obtained with the design of the experiment, two optimized NLC formulations, one with low (30 nm) and another with high particle size (85 nm) were produced. Both optimized formulations were characterized by low PDI, high encapsulation efficiency and were physically and chemically stable for over 30 days. XDR and DSC analyses were excellent tools for the characterization of the crystal order the lipid in the formulations and showed that NLC evidenced a decrease in the crystallinity index when compared Ucuùba fat. The total absence of the ketoconazole signals in the DSC thermograms and XDR spectra, suggested an optimal drug encapsulation in the lipid matrix and no sign of drug expulsion was evidenced.

The optimized formulations obtained will be further investigated for their antifungal efficacy against onychomycosis.

## Figures and Tables

**Table 1:** Input factors and their levels for Box-Behnken design.

Input Factors	Levels		
	−1	0	+1
Surfactant Concentration (% *w*/*v*, *X*_1_)	3	4.5	6
Liquid Lipid Concentration (% *w*/*v*, *X*_2_)	2	2.5	3
Solid Lipid Concentration (% *w*/*v*, *X*_3_)	7	7.5	8

**Table 2:** Experimental results of the Box-Behnken design.

Experiment #	Surfactant (% *w*/*v*)	Liquid Lipid (% *w*/*v*)	Solid Lipid (% *w*/*v*)	Size (nm)	*PDI* ^1^	Ketoconazole *EE* (%)
1	4.5	2	8	65.9	0.506	93.91
2	3	2.5	8	79.4	0.248	98.71
3	6	2.5	7	26.6	0.432	96.05
4	4.5	3	8	68.0	0.413	97.16
5	4.5	3	7	45.1	0.492	99.66
6	4.5	2.5	7.5	45.4	0.360	97.75
7	3	2	7.5	82.1	0.291	94.67
8	3	3	7.5	90.1	0.311	97.26
9	4.5	2.5	7.5	47.6	0.399	97.67
10	6	2.5	8	39.8	0.533	97.99
11	6	2	7.5	66.8	0.558	96.38
12	4.5	2.5	7.5	43.3	0.449	98.56
13	4.5	2	7	54.0	0.481	97.95
14	4.5	2.5	7.5	48.1	0.329	99.50
15	3	2.5	7	69.8	0.263	96.55
16	6	3	7.5	23.9	0.288	98.29
17	4.5	2.5	7.5	63.0	0.461	96.57

^1^ Polydispersity Index (*PDI* ≤ 0.1 highly monodisperse particles; 0.1–0.4 slightly polydisperse; ≥ 0.4 highly polydisperse samples) [34].

**Table 3:** ANOVA results obtained for the regression model calculated from the experimental design data for the prediction particle size, *PDI* and *EE*.

Factors	Particle Size (*Y*_1_)			*PDI* (*Y*_2_)			*EE* (*Y*_3_)		
	Coefficient Estimate	*F*-Value	*p*-Value	Coefficient Estimate	*F*-Value	*p*-Value	Coefficient Estimate	*F*-Value	*p*-Value
Intercept	49.49	11.56	0.0020 *	0.3996	4.26	0.0346 *	98.1	0.64	0.7392
*X*_1_	−20.54	66.07	0.0001 *	0.0873	17.58	0.0041 *	0.1900	0.10	0.7649
*X*_2_	−5.21	4.24	0.0784	−0.0415	3.98	0.0864	1.18	3.74	0.0942
*X*_3_	7.19	8.10	0.02 *	0.0040	0.04	0.8530	−0.3050	0.25	0.6330
*X*_1_ *X*_2_	−12.73	12.69	0.0092 *	−0.0725	6.07	0.0433 *	−0.1700	0.04	0.8497
*X*_1_ *X*_3_	0.8825	0.06	0.8120	0.0290	0.97	0.3573	−0.0550	0.00	0.9510
*X*_2_ *X*_3_	2.76	0.59	0.4660	−0.0260	0.78	0.4063	0.3850	0.20	0.6695
$X_{1}^{2}$	5.96	2.93	0.1307	−0.0708	6.09	0.0430 *	−0.6025	0.51	0.4976
$X_{2}^{2}$	10.29	8.73	0.0212 *	0.0332	1.34	0.2851	−0.7575	0.81	0.3984
$X_{3}^{2}$	−1.56	0.20	0.6682	0.0332	1.96	0.2038	−0.0825	0.01	0.9247
**Model**									
***R*^2^**		0.9369			0.8456			0.4513	
***F*-value**		11.48			4.33			0.6347	

* Significant effect of the factor on individual responses. Abbreviations: ANOVA, analysis of variance; *PDI*, polydispersity index; *EE*, encapsulations efficiency; *X*_1_ surfactant concentration; *X*_2_ liquid lipid concentration; *X*_3_ solid lipid concentration; *F*-value, ratio of the mean regression sum of squares divided by the mean error sum of squares; *R*^2^, coefficient of determination.

**Table 4:** Composition of the optimized Ucuùba fat NLC formulations F 30 and F 85 along with the predicted, observed and residual values of the responses.

Input Factors	F 30			F 85		
Surfactant (% *w*/*v*)	6			3		
Liquid Lipid (% *w*/*v*)	3			2.75		
Solid Lipid (% *w*/*v*)	7.63			7.68		
Responses	Predicted	Observed	Residual *	Predicted	Observed	Residual *
Size (nm)	30.0	33.6 ± 0.2	−3.6	85.0	74.6 ± 0.3	10.4
*PDI*	0.339	0.255 ± 0.001	0.084	0.257	0.143 ± 0.005	0.114
*EE* (%)	97.85	98.20 ± 0.62	−0.35	97.67	98.70 ± 0.69	−1.03

* Residual = Predicted − Observed.

**Table 5:** Physico-chemical characterization of optimized Ucuùba fat NLC during storage at room temperature.

Time (days)	F 30			F 85		
	Z-Average (nm)	*PDI*	Zeta Potential (mV)	Z-Average ^1^ (nm)	*PDI*	Zeta Potential (mV)
0	33.6 ± 0.2	0.26 ± 0.01	−15.2 ± 0.8	74.6 ± 0.3	0.14 ± 0.00	−24.5 ± 0.6
1	33.7 ± 0.1	0.20 ± 0.01	−16.4 ± 1.3	74.1 ± 0.2	0.13 ± 0.02	−21.3 ± 1.1
7	34.4 ± 0.3	0.19 ± 0.00	−13.3 ± 1.1	72.9 ± 0.3	0.16 ± 0.04	−20.2 ± 6.5
15	33.3 ± 0.2	0.22 ± 0.01	−12.1 ± 1.3	71.1 ± 0.1	0.15 ± 0.01	−17.4 ± 0.6
30	33.8 ± 0.2	0.20 ± 0.01	−15.2 ± 0.8	73 ± 0.2	0.15 ± 0.01	−20.2 ± 1.5

^1^ Z-average = Average particle size.

**Table 6:** Melting temperatures, enthalpy and crystallinity index (*CI*) obtained by DSC analysis of the Ucuùba fat alone and in mixture with Capryol™ 90.

Ucuùba Fat/Capryol™ 90 Weight Ratio	*T*_onset_ (°C)	*T*_endset_ (°C)	Peak (°C)	Enthalpy (J/g)	*CI* (%)
1:0	37.76	45.11	42.99	−99.42	100
3:1	31.96	41.77	39.79	−71.38	53.84
1:1	28.90	40.00	36.98	−52,01	26.15
1:2	26.61	38.91	35.24	−26.09	8.72
